# Pediatric epilepsy surgery in patients with Lennox-Gastaut syndrome after viral encephalitis

**DOI:** 10.3389/fneur.2023.1097535

**Published:** 2023-02-24

**Authors:** Qingzhu Liu, Nan Wu, Chang Liu, Hao Yu, Yu Sun, Yao Wang, Guojing Yu, Shuang Wang, Taoyun Ji, Xiaoyan Liu, Yuwu Jiang, Lixin Cai

**Affiliations:** ^1^Pediatric Epilepsy Center, Peking University First Hospital, Beijing, China; ^2^Department of Neurosurgery, Tianjin Children's Hospital, Children's Hospital of Tianjin University, Tianjin, China; ^3^Department of Pediatrics, Peking University First Hospital, Beijing, China

**Keywords:** Lennox-Gastaut syndrome, viral encephalitis, epilepsy surgery case management, epilepsy surgery–catastrophic epilepsy, viral encephalitis in children

## Abstract

**Objective:**

To analyse the surgical outcomes of pediatric patients with Lennox-Gastaut syndrome (LGS) secondary to viral encephalitis.

**Methods:**

We retrospectively analyzed the data of four patients with LGS secondary to viral encephalitis who underwent surgery at the pediatric epilepsy center of Peking University First Hospital from January 2014 to December 2019. Preoperative evaluations included a detailed history, long-term video electroencephalography (VEEG), brain magnetic resonance imaging (MRI), positron emission tomography (PET) and a neuropsychological test. All patients were followed up at 1, 3, and 6 months and then yearly. The surgical outcome was evaluated according to the Engel classification.

**Results:**

Among the four children, the surgeries were right temporo-parieto-occipital disconnection (case 1), corpus callosotomy (case 2), left temporo-parieto-occipital disconnection (case 3), and left temporal lobectomy (case 4). The pathology was gliosis secondary to viral encephalitis. The median follow-up time was 4 years (3–5 years). At the last follow-up, one case had Engel I, two cases had Engel III, and one case had Engel IV.

**Conclusions:**

Preliminary observations shows that surgical treatment may be challenging for patients with LGS secondary to viral encephalitis. However, suitable surgical candidacy and approaches have a significant impact on the prognosis of the patients.

## 1. Introduction

Lennox-Gastaut syndrome (LGS) is considered an epileptic encephalopathy and is characterized by a triad of intractable seizures (in particular tonic seizures during sleep, but atonic and atypical absence seizures are also common), cognitive and behavioral impairments and diffuse slow spike-and-wave (SSW) and paroxysms of fast activity (PFA) on electroencephalography (EEG) (1). LGS is estimated to affect between 1 and 2% of all patients with epilepsy (2). Generally, LGS often occurs in young children (3).

The etiology of LGS can be classified as cryptogenic or symptomatic. LGS of symptomatic etiology may be secondary to hypoxic ischemic encephalopathy, congenital brain malformation, vascular malformation, genetic conditions such as tuberous sclerosis, trauma, brain tumor, or perinatal meningoencephalitis (4). Among them, viral encephalitis (VE) is a known cause of LGS. VE is a severe disease of the central nervous system that usually presents with seizure attacks and progressive neurological deficits in the acute phase (5, 6). Intractable epilepsies that occur several months to years after acute encephalitis are called postencephalitis epilepsies (PEs), some of which present as LGS (7, 8).

Although the majority of patients with LGS have diffuse EEG patterns, some focal changes causing secondary generalized epileptic encephalopathy can be identified on neuroimaging (1). Patients with such focal lesions can be treated surgically through comprehensive preoperative evaluation. However, studies on the surgical treatment of LGS after VE are rare. Therefore, the objective of this study was to discuss surgical strategies for secondary LGS of VE origin in children.

## 2. Materials and methods

### 2.1. Methods

Between January 2014 and December 2019, we retrospectively collected the data from four children with LGS secondary to viral encephalitis who underwent surgery at the pediatric epilepsy center of Peking University First Hospital. All patients were young boys, with a median age of 6 years (6–9 years). The diagnostic criteria for encephalitis are an altered mental status lasting more than 24 h, with at least three minor criteria (9): (1) body temperature ≥38°C within 72 h, (2) generalized or partial seizures, (3) focal neurologic findings, (4) cerebral spinal fluid (CSF) white blood count ≥5/cubic mm, (5) abnormal brain parenchyma on neuroimaging, and (6) abnormalities on EEG. Moreover, on CSF analysis, if the patient had a positive virus antibody titer, they were considered to have VE. Children with bacterial meningitis, post vaccination encephalitis, and Rasmussen encephalitis were excluded. This study was approved by the Institutional Review Board of the Ethics Committee of Peking University First Hospital.

### 2.2. Preoperative evaluation

All patients underwent a comprehensive preoperative evaluation, including a detailed historical investigation, long-term video EEG (VEEG), brain magnetic resonance imaging (MRI), positron emission tomography (PET) and a neuropsychological test. Brain 3.0 T MRI scans were performed, including T1, T2, and fluid-attenuated inversion recovery (FLAIR) sequences. Coregistration between PET and MR images was performed to identify potential lesions and improve the resolution of the localization.

### 2.3. Operation

All patients underwent surgery under general anesthesia. The surgical incision was designed according to the preoperative evaluation results. Before electrocorticogram monitoring, the amount of propofol administered was reduced to monitor epileptiform discharges. Resection and disconnection under the pia mater were performed.

### 2.4. Postsurgical outcome

According to the Engel classification, the seizure outcomes of the children were evaluated at our center 1, 3, and 6 months postoperatively and then yearly. Functional outcome was evaluated by physical examinations, including those for muscle strength, muscle tension and language function (understanding, reading, etc.).

## 3. Results

### 3.1. Clinical features

**Figure 1:**
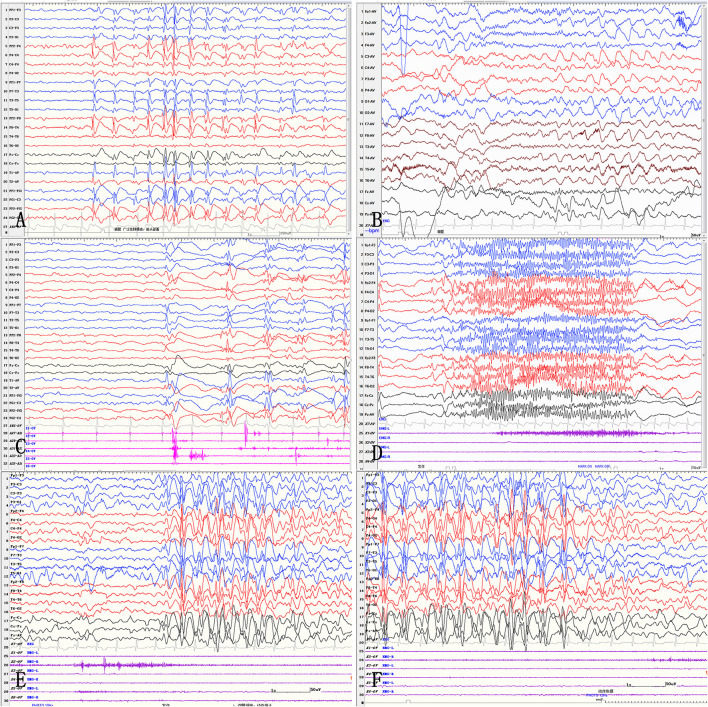
Interictal and ictal EEG in LGS patients. Interictal EEG shows generalized 2.5–3 Hz medium-high amplitude spike and slow waves, mainly located in the bilateral frontal regions **(A)**. Paroxysms of fast activities are shown on interictal EEG **(B)**. Myoclonic seizures show generalized sharp and slow waves **(C)**. Tonic seizures show generalized low-amplitude rhythms of spike waves **(D)**. Atypical absence seizures show diffuse, 2.5–3 Hz medium-high amplitude spike waves and spike-and-slow wave discharges **(E, F)**.

The clinical data, including the seizure types, of the patients are summarized in Table 1. The history of febrile convulsions was presented in cases 2, 3, and 4. All patients had normal immune state and excluded autoimmune encephalitis. All patients presented with tonic seizures and spasm. Before surgery, the physical examinations revealed that muscle strength and muscle tension were normal for cases 1, 3, and 4. The patient in case 2 had mild hemiparesis in his right limbs. The characteristics of the interictal EEG often showed diffuse SSW and PFA, and there was no obvious lateralization on ictal EEG (Figure 1).

**Table 1:** Clinical, radiological, and surgical profiles.

**Case**	**Age at onset**	**Age at surgery**	**Seizure types**	**Seizure frequency**	**Scalp EEG findings**		**Neuroimaging abnormalities**		**Surgery**	**FU**	**Seizure OC**	**NPMD**
					**Interictal**	**Ictal**	**MRI**	**PET**				
1	1y3m	6y3m	Tonic, Atypical absence, Myoclonic, Spasm	1-2 times/day	Diffuse SSW, PFA,	Diffuse	Ence in Rt-T, Ab in Rt-PO	Hypo in Rt-TPO	Rt-TPO	80m	Ia	No
2	1y	9y	Tonic, Myoclonic, Spasm	>10 times/day	Diffuse SSW, predominantly in the B-F region	Diffuse	Ab in B-FPT	NA	CC	60m	III	No
3	1y	5y5m	Tonic, Atypical absence, Spasm	3-4 times/day	Diffuse SSW, PFA	Diffuse	Ence in Lt-TPI, Ab in Rt-I	Hypo in Lt-TPI	Lt-TPO	50m	III	No
4	3y	6y10m	Tonic, Atonic, Atypical absence, Myoclonic, Spasm	>10 times/day	Diffuse SSW, predominantly in the Lt-T region	Diffuse	Ence in Lt T	Hypo in Lt TFP	Lt-T	38m	IV	No

EEG, electroencephalography; MRI, magnetic resonance imaging; PET, positron emission computed tomography; FU, follow up; OC, outcome; NPMD, new permanent motor deficit; y, year; m, month; SSW, slow spike-and-wave; PFA, paroxysms of fast activities; Ence, encephalomalacia; Rt, right; Lt, left; B, bilateral; Ab, abnormal signal; T, temporal; P, parietal; O, occipital; F, frontal; I, insular; Hypo, hypometabolism; NA, not available; CC, corpus callosotomy.

**Figure 2:**
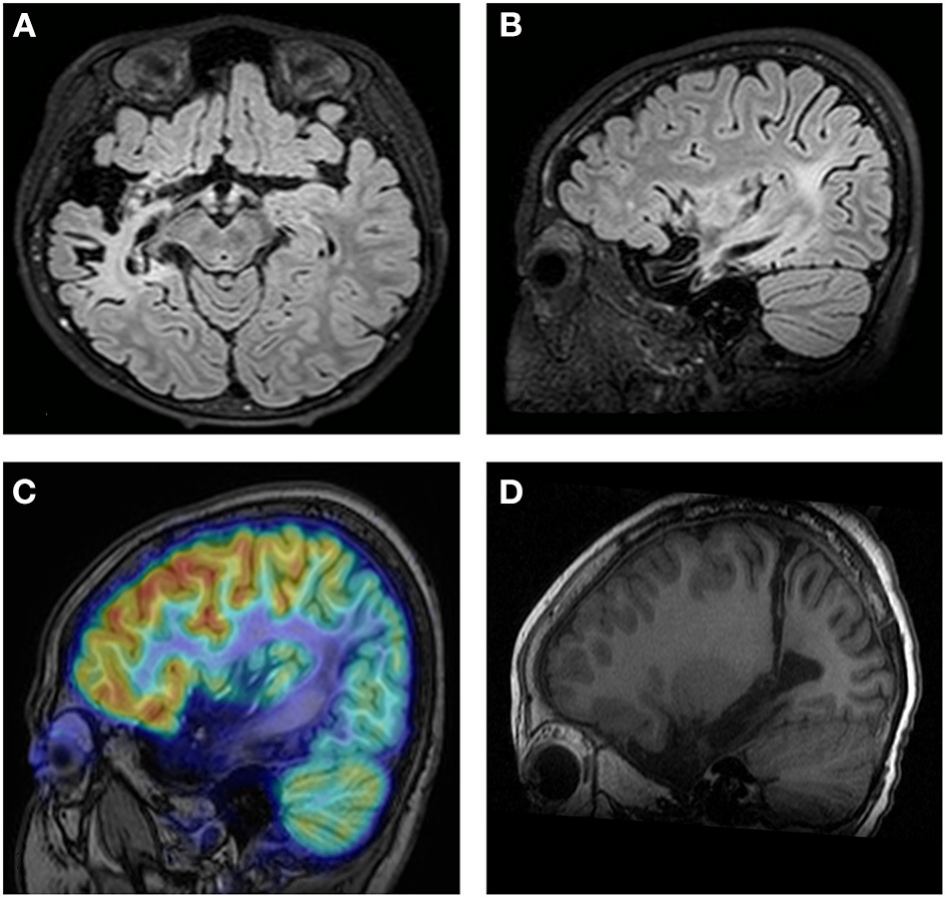
Neuroimaging data of case 1. MRI reveals encephalomalacia in the right temporal lobe **(A)**. Abnormal signals are observed in the right parietal and occipital lobes **(B)**. PET-MRI Coregistration shows hypometabolism in the temporal, parietal and occipital lobes **(C)**. Postoperative MRI shows disconnection of the temporo-parieto-occipital lobes **(D)**.

**Figure 3:**
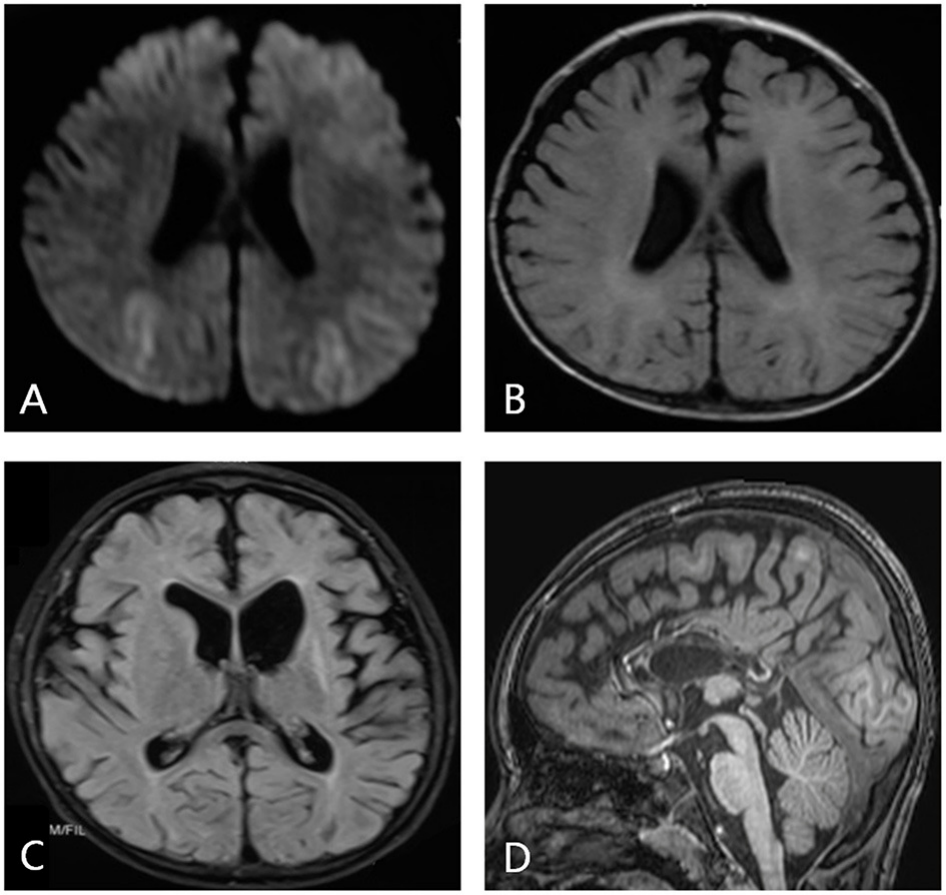
Neuroimaging data of case 2. During the acute phase of viral encephalitis, MRI shows an abnormal signal in the bilateral frontal and parietal regions on diffusion-weighted imaging **(A)** and T1-weighted imaging **(B)**. MRI reveals a more obvious signal on the left side, and dilation of the left lateral ventricle is observed **(C)**. Imaging shows completed corpus callosotomy **(D)**.

**Figure 4:**
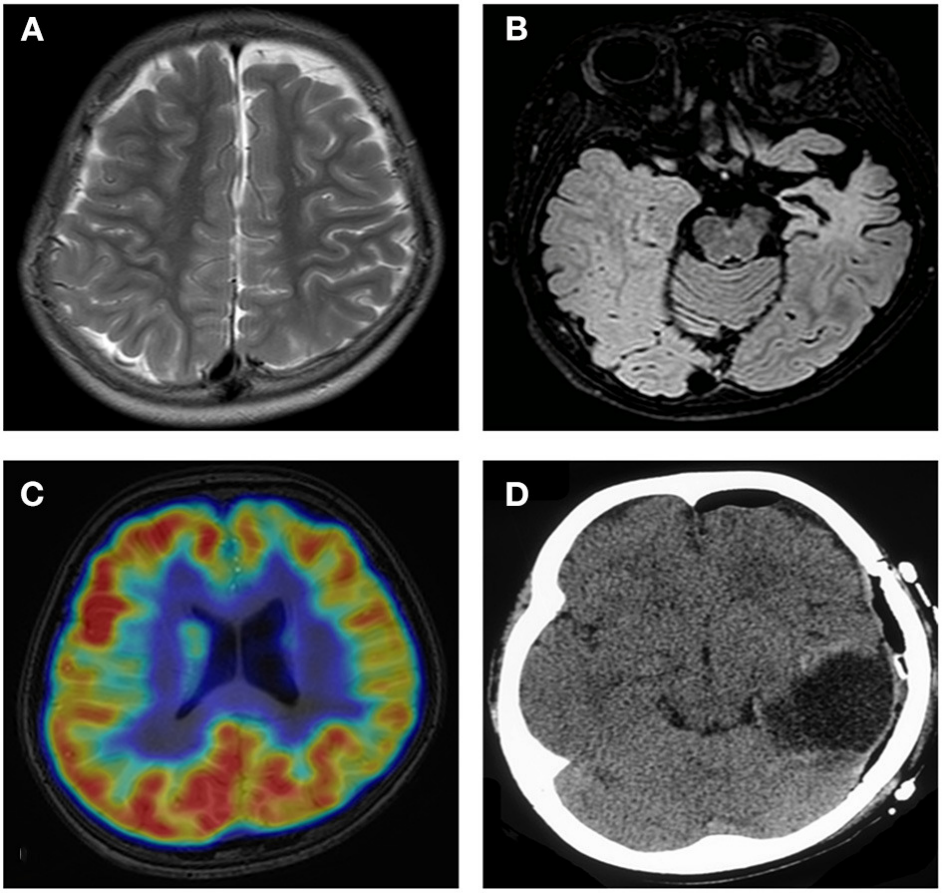
Neuroimaging data of case 4. MRI shows mild atrophy in the left hemisphere **(A)**. MRI reveals encephalomalacia in the left temporal lobe **(B)**. PET shows hypometabolism in the frontal and parietal lobes **(C)**. Imaging shows completed left temporal lobectomy **(D)**.

Obvious lesions were observed in the neuroimaging results. In case 1, MRI revealed encephalomalacia located in the right temporal lobe, while abnormal signals were observed in the right parietal and occipital lobes (Figure 2). In case 2, multiple abnormal signals were located bilaterally, with more obvious lesions appearing on the left side (Figure 3). In case 3, MRI showed encephalomalacia mainly in the left temporal, parietal and insular lobes, while mildly abnormal signal was located in the right posterior insular lobe. In case 4, MRI revealed encephalomalacia in the left temporal lobe and mild atrophy in the left hemisphere (Figure 4). PET was performed for three patients (cases 1, 3, and 4). In cases 1 and 3, the hypometabolism seen on PET was concordant with the lesions observed on MRI. In case 4, the extent of hypometabolism on PET was larger than that on MRI.

### 3.2. Surgical data

The four patients underwent different surgical approaches (Table 1). Right temporo-parieto-occipital (TPO) disconnection was performed in case 1, corpus callosotomy (CC) in case 2, left TPO disconnection in case 3, and left temporal lobectomy in case 4. There were no postoperative complications, such as intracranial infection, cerebral hemorrhage or infarction. However, relative to baseline, postoperative transient motor deficits were observed in two children (Cases 1 and 3), who recovered completely within 2–4 weeks. A new permanent motor deficit was defined as a new motor functional deficit that could no longer be reversed 3 months postoperatively. There was no worsening postoperative language dysfunction any of the four children. Due to the young ages and noncooperation of the children in our cohort, we did not perform neuro-ophthalmological examinations for hemianopia before/after surgery.

The median follow-up time of the 4 patients was 4 years (3–5 years). At the last follow-up, one patient had Engel I (Case 1), two had Engel III (Cases 2 and 3), and 1 had Engel IV (Case 4).

Case 1 was subjected to right TPO disconnection. Postoperative EEG showed high amplitude spikes, polyspikes, and fast activities mainly located in the right TPO region. The patient was seizure free at the last follow-up.

CC was performed for Case 2, but the seizures recurred 9 days postoperatively, presenting as spasms and myoclonic seizures without tonicity. Postoperative EEG showed spikes and spike-and-slow wave complexes located in the bilateral frontal, parietal, and temporal regions, predominantly on the left side. At the last follow-up, more than 80% seizure reduction was reported. Both intellectual and motor development were improved postoperatively.

In case 3, left TPO disconnection was performed. Seizures recurred 1 year postoperatively, presenting as spasms without the presence of tonic or atypical absence seizures. Postoperative EEG revealed a sharp wave located in the left parietal, occipital, and posterior temporal regions. Fast activities and polyspikes were located in the left frontal and central regions and right posterior temporal and parietal regions.

Left temporal lobectomy was performed for Case 4. The same types of seizure recurred 1 month postoperatively. Postoperative EEG showed spike waves and slow-spike waves in all montages, predominantly in the left posterior temporal region.

## 4. Discussion

Lennox-Gastaut syndrome is one of the most serious forms of intractable epilepsy in children. Generally, during the course of LGS, more than 70% of patients show no response to anti-seizure medicines, and less than 10% of patients achieve a seizure-free status (10). It has been reported in the literature that for LGS children with focal lesions on MRI, despite a lack of localized epileptic patterns on EEG, epilepsy surgery may be a treatment strategy (11, 12).

Malformation during cortical development is the most common cause of surgeries for children with LGS; other reasons include tuberous sclerosis complex, perinatal complications, craniocerebral trauma, and infection (13). The reported risk of late unprovoked seizures in population-based cohorts of cerebral infection survivors from developed countries is estimated at 7–8% (5). Studies have reported that the main risk factors for epilepsy after VE include epileptic seizures in the acute phase of encephalitis, status epilepticus, and abnormally hyperintense T2/FLAIR signals on MRI, often involving the temporal and frontal lobes (14, 15).

Children with LGS secondary to VE often present with a number of unique characteristics. The diagnosis of LGS is much more complicated in the clinic. The inclusion criteria included seizures, particularly generalized tonic, atonic and myoclonic seizures as well as atypical absence seizures and spasms, generalized SSW and PFA on EEG and progressive developmental regression (16). However, it can be difficult to find all the conditions that fulfill the diagnostic criteria. Moreover, patients with LGS secondary to VE often have obvious structural brain lesions on MRI, despite diffuse or generalized epileptiform discharge patterns on EEG. Discordance in the investigations may lead to more difficult localization of the epileptogenic zone.

Despite the high prevalence of drug-resistant epilepsies after cerebral infection, especially in patients with MRI-identifiable lesions, only a small number of patients undergo epilepsy surgery (14). The effect of surgical treatment of epilepsy secondary to VE is often poor because of the large extent of brain damage during acute VE, which often involves bilateral brain regions. In the acute phase, MRI is often inconsistent with that in the residual phase. However, the surgical outcomes in certain subgroups of patients with epilepsy after VE are encouraging (17). At present, there is no literature reporting the surgical outcomes of patients with LGS secondary to VE.

Among our cases, all of our patients had obvious lesions on MRI, despite the lack of localized epileptic patterns on interictal and ictal EEG. These patients all underwent different surgical approaches, and the prognoses were different. The surgical strategies were mainly based on neuroimaging data.

If the MRI shows obvious bilateral lesions, such as in case 2 in our series, palliative surgery should be performed. Both CC and vagus nerve stimulation (VNS) are safe and effective treatments for LGS patients (18, 19). Some meta-analyses have supported that CC has a better seizure outcome than VNS (20, 21). Therefore, in our case, CC was performed, and a more than 80% seizure reduction was reported. Both intellectual and motor development were improved postoperatively, which is consistent with previous literature (22–24).

If instead, MRI reveals obvious unilateral lesions, as in cases 1, 3, and 4 in our series, curative surgery may be a promising treatment. However, some important tips should be noted. First, although the three cases presented with prominent lesions on one side, mild abnormalities can sometimes be observed on the contralateral side. Surgery on the prominent side is intended to reduce the frequency and severity of seizure attacks. In case 3, left TPO disconnection was performed, but a mild abnormal signal was observed in the right posterior insular lobe. Seizures recurred 1 year postoperatively, but tonic and atypical absence seizures were not observed. Therefore, in these situations, the surgeon and parents should have extensive discussions regarding the advantages and disadvantages of surgery. Second, MRI in the residual phase should be compared with that performed in the acute phase. Sometimes, more obvious lesions can be seen in FLAIR/diffusion-weighted imaging in the acute phase, while in the residual phase, there are no obvious lesions in the same region on MRI.

Although MRI provides much more localization or lateralization information, PET images can provide supplementary information for the former. In some studies, inconsistency between PET and MRI often heralded a poor outcome (17, 25). In case 4, MRI revealed encephalomalacia in the left temporal lobe; however, PET showed hypometabolism that was more extensive than the encephalomalacia observed on MRI. Left temporal lobectomy was performed, but the seizures recurred 1 month postoperatively. Therefore, PET should be carefully analyzed to better determine the border of the lesions.

The medical history should be noted as well. Cases 2, 3 and 4 had a history of febrile convulsions in the acute phase. And all of them had poor outcome. However, previous studies found that febrile convulsions were a predictor of good surgical outcome (26, 27). Different etiology may explain this apparent discrepancy. Our study included only patients with LGS secondary to VE. Previous studies included patients with mesial temporal lobe epilepsy secondary to hippocampal sclerosis. Moreover, immune state also should be noted. Anti-N-methyl-D-aspartate receptor (NMDAR) encephalitis after virus infection is a recently identified constellation that should be distinguished from drug-resistant epilepsy secondary to VE. NMDAR antibodies have been frequently detected in patients with virus encephalitis. Anti-NMDAR encephalitis after virus infection often received immunosuppressive therapy instead of surgery, because surgical treatment for anti-NMDAR encephalitis after virus infection often associated with poor outcome.

There are several limitations in our study. This was a retrospective study, and only four cases were included; therefore, statistical analysis could not be performed. More patients will be included in further investigations to analyse the prognostic factors for the outcome of treatments for LGS.

## 5. Conclusions

Surgery is a challenging treatment method for patients with LGS secondary to VE. The surgical outcomes can differ due to anatomo-electro-clinical correlations. Generally, compared to malformation of cortical development, VE results in more obvious lesions on MRI. However, it should be emphasized that careful analysis of MRI features of MRI in both the acute and residual phases is very crucial. Concordance between MRI lesions and PET imaging findings corresponded to better surgical outcomes. In addition, generalized seizures and diffuse scalp EEG findings were not contraindications to epilepsy surgery. Therefore, surgical decisions regarding LGS secondary to VE should be determined very cautiously.

## Data availability statement

The raw data supporting the conclusions of this article will be made available by the authors, without undue reservation.

## Ethics statement

This study was approved by the Institutional Review Board of the Ethics Committee of Peking University First Hospital. Written informed consent to participate in this study was provided by the participants' legal guardian/next of kin. Written informed consent was obtained from the individual(s), and minor(s)' legal guardian/next of kin, for the publication of any potentially identifiable images or data included in this article.

## Author contributions

LC, YJ, and XL designed the study and revised the paper. NW, CL, and QL analyzed the data and drafted and revised the paper. CL, HY, and YS collected the data. SW and TJ helped to select the patients. GY helped to interpret the EEG data. YW performed the patient follow-up. All authors contributed to the article and approved the submitted version.
